# Gen2Epi: an automated whole-genome sequencing pipeline for linking full genomes to antimicrobial susceptibility and molecular epidemiological data in *Neisseria gonorrhoeae*

**DOI:** 10.1186/s12864-019-5542-3

**Published:** 2019-03-04

**Authors:** Reema Singh, Jo-Anne R. Dillon, Walter Demczuk, Anthony Kusalik

**Affiliations:** 1 Department of Biochemistry Microbiology and Immunology, 2D01 Health Science Building, 107 Wiggins Road, University of Saskatchewan, Saskatoon, SK S7N5E5 Canada; 2 Vaccine and Infectious Disease Organization-International Vaccine Centre, University of Saskatchewan, 120 Veterinary Road, Saskatoon, SK S7N5E3 Canada; 3 Public Health Agency of Canada, National Microbiology Laboratory, Winnipeg, MB Canada; 4 Department of Computer Science, University of Saskatchewan, 176 Thorvaldson Building, 110 Science Place, University of Saskatchewan, Saskatoon, SK S7N5C9 Canada

**Keywords:** Bioinformatics, Whole-genome sequencing (WGS), De novo genome assembly, Scaffolding, Molecular epidemiology, Strain typing, Antimicrobial resistance, Molecular typing, *Neisseria gonorrhoeae*

## Abstract

**Background:**

Recent adva1nces in whole genome sequencing (WGS) based technologies have facilitated multi-step applications for predicting antimicrobial resistance (AMR) and investigating the molecular epidemiology of *Neisseria gonorrhoeae*. However, generating full scaffolds of *N. gonorrhoeae* genomes from short reads, and the assignment of molecular epidemiological information (NG-MLST, NG-MAST, and NG-STAR) to multiple assembled samples, is challenging due to required manual tasks such as annotating antimicrobial resistance determinants with standard nomenclature for a large number of genomes.

**Results:**

We present Gen2Epi, a pipeline that assembles short reads into full scaffolds and automatically assigns molecular epidemiological and AMR information to the assembled genomes. Gen2Epi is a command-line tool integrating third-party software and tailored specifically for *N. gonorrhoeae*. For its evaluation, the Gen2Epi pipeline successfully assembled the WGS short reads from 1484 *N. gonorrhoeae* samples into full-length genomes for both chromosomes and plasmids and was able to assign in silico molecular determinant information to each dataset automatically. The assemblies were generated using raw as well as trimmed short reads. The median genome coverage of full-length scaffolds and “N” statistics (N50, NG50, and NGA50) were higher than, or comparable to, previously published results and the scaffolding process improved the quality of the draft genome assemblies. Molecular antimicrobial resistant (AMR) determinants identified by Gen2Epi reproduced information for the 1484 samples as previously reported, including NG-MLST, NG-MAST, and NG-STAR molecular sequence types.

**Conclusions:**

Gen2Epi can be used to assemble short reads into full-length genomes and assign accurate molecular marker and AMR information automatically from NG-STAR, NG-MAST, and NG-MLST. Gen2Epi is publicly available under “CC BY-NC 2.0 CA” Creative Commons licensing as a VirtualBox image containing the constituent software components running on the LINUX operating system (CentOS 7). The image and associated documentation are available via anonymous FTP at ftp://www.cs.usask.ca/pub/combi or ftp://ftp.cs.usask.ca/pub/combi

**Electronic supplementary material:**

The online version of this article (10.1186/s12864-019-5542-3) contains supplementary material, which is available to authorized users.

## Background

Gonorrhea, a sexually transmitted infection (STI) caused by *Neisseria gonorrhoeae* (Ng), is a global public health problem, magnified by high levels of resistance to antimicrobial agents [1]. The introduction of whole-genome sequencing (WGS) technologies has allowed the tracking of gonococcal transmission and resistance to all classes of available antimicrobials [2]. In order to manage and analyze the large amount of data generated from sequencing techniques, initiatives to develop appropriate tools and software have been undertaken by the *N. gonorrhoeae* scientific community worldwide [3] with a number of methods developed to assemble genomes, predict antimicrobial resistance (AMR), and investigate strain transmission through analysis of genomic molecular determinants.

A variety of bacterial strain typing facilities have been made available to the community. Jolly and Maiden initially developed BIGSdb (Bacterial Isolate Genome Sequence Database) [4] to store and investigate Multilocus Sequence Typing (MLST) profiles of sequences generated from multiple sources, such as multiple single amplicons or assembled contigs from sequencing technologies. The BIGSdb database was supplanted by pubMLST [5], which has been subsequently used by the scientific community to store and exploit WGS data for strain characterization. The web-based components of the pubMLST database [6] were implemented using MLST software [7]. Inouye et al. developed SRST [8] (Short Read Sequence Typing) software implemented for a number of bacteria (including *N. gonorrhoeae*) to determine sequence/strain type directly from short read WGS data. Molecular strain typing schemes for *N. gonorrhoeae* have also been developed based on molecular antimicrobial resistance determinants, such as the web-based NG-STAR (*Neisseria gonorrhoeae* Sequence Typing for Antimicrobial Resistance) [9] typing scheme. Although this web-based application allows batch upload of AMR gene sequences from multiple samples, bulk download of annotated antimicrobial resistance determinants is a manual process [10]. A third typing procedure, called NG-MAST (*N. gonorrhoeae* Multiantigen Sequence Typing), is based on the sequences of two highly variable genes, *porB,* and *tbpB* [11]. Martin et al. [12] developed a publicly available database of sequences of *porB* and *tbpB* alleles, which was utilized to build a command-line software tool named NGMASTER to assign NG-MAST strain types to whole genome assemblies [13]. All these individual typing schemes give a comprehensive picture of *N. gonorrhoeae* strain characterization; however, until the present, there has been no publicly available tool or software available to consolidate these various typing schemes at one place.

Several experiments describe the use of WGS assemblies to understand the molecular epidemiology of gonococcal isolates [14–18], including recently published Australian [15], New Zealand [14] and EuroGASP 2013 [18] studies. These analyses [14–16] terminated the *N. gonorrhoeae* WGS assembly process at the level of contigs. A disadvantage of this approach is the possibility of missing a gene during gene identification or making an error in the number of predicted genes due to gene fragmentation over multiple contigs. Despite the presence of scaffolding tools, none of the previously published WGS studies assembled contigs into full genomes, except in one study where authors used PacBio long reads to assemble *N. gonorrhoeae* World Health Organization (WHO) reference strains [17], and a second study recently published by Harris et al. [18], which used SPAdes [19] along with the Sanger Institute assembly pipeline [20] to assemble short reads into scaffolds. The entire dataset generated by the European genomic survey [18] has been made available at the Pathogenwatch website [21]. The website allows users to access full genomes from different bacterial species along with investigative methods such as MLST analysis, AMR predictions, genome clustering, and interactive visualization. However, a shortcoming of this web application [21] is the lack of important analysis steps such as data cleaning, de novo assembly, scaffolding, and annotation.

There have been a number of methods and pipelines developed to assemble bacterial contigs into scaffolds; for instance, Ragout [22], SSPACE [23], SSPACE-LongRead [24], and ALLPATHS [25]. The Sanger Institute assembly pipeline [20] improves assembly by joining assembled contigs into scaffolds but it lacks further downstream analysis such as annotation, strain typing, and AMR prediction.

In published studies for *N. gonorrhoeae*, different assemblers, pipelines, and web applications have been utilized to analyze the genomes [14, 26–28]. Among the frequently used techniques are SPAdes [19], Velvet [26, 27], and CLC Workbench [28]. Another commonly used method in the *Neisseria* community is the Nullarbor pipeline, which performs complete analysis, from read cleaning to variant calling [29]. However, it lacks NG-MAST and NG-STAR typing schemes.

Despite the advances in WGS techniques, generating full scaffolds of *N. gonorrhoeae* genomes from assembled short reads, and the assignment of in silico molecular marker information to multiple samples, is challenging due to required, typically manual steps, such as annotating antimicrobial resistance determinants with the standard nomenclature for a large number of genomes. Here, we present a one-step computational WGS pipeline, named Gen2Epi, to 1) assemble short read datasets into full-length genomes, and 2) assign molecular epidemiological and AMR designations to the assembled genomes automatically. We assessed the performance of the Gen2Epi pipeline by using Illumina short reads from 1484 whole genome sequencing samples from previously published studies [14, 17, 18, 30, 31] and also determined the accuracy by comparing to previously reported results [14, 17, 18, 30, 31]. Our long-term goal is to make this a user-friendly pipeline readily accessible to public health researchers not familiar with in-depth bioinformatics approaches.

## Implementation

### Data used

The Gene2Epi pipeline was tested using 1484 *N. gonorrhoeae* sequencing-read datasets from previously published studies (Additional file 1: Table S1) [14, 17, 18, 30, 31]. The first study included WHO *N. gonorrhoeae* reference strains (WHO F, G, K, L, M, N, O, P, X, Y, and Z) [17]. Full reference genomes of these strains were available at the European Nucleotide Archive (ENA) [32] (Additional file 1: Table S1). The second study included 27 isolates that originated from patients in Saskatchewan, Canada [30, 31]. The third and fourth studies included 398 *N. gonorrhoeae* datasets from New Zealand [14] and 1048 isolates from the EuroGASP 2013 collection [18] downloaded from National Center for Biotechnology Information (NCBI) [33]. Six samples were excluded from the 1054 WGS samples in the original EuroGASP study (see Additional file 1: Table S1 for more information). WGS data available from all these studies were generated on Illumina platforms (Additional file 1: Table S1). NCCP11945 and FA1090 *N. gonorrhoeae* reference genomes were downloaded from the NCBI [33]. Sequences of known *N. gonorrhoeae* plasmids (see Additional file 1: Table S2 for references) and NG-MLST and NG-STAR genes (Additional file 1: Table S2) were downloaded from the NCBI public nucleotide repository [33]. MLST allelic sequences and profiles were retrieved from pubMLST (July 4, 2018) [6]. FASTA sequences of NG-STAR [7] allele sequences, as well as profile and metadata associated with seven AMR genes, were downloaded from the NG-STAR website (July 4, 2018) [10].

During Gen2Epi testing, a number of reference genomes were utilized for the scaffolding process. For instance, available WHO reference genomes were used in the case of WHO datasets [17], *N. gonorrhoeae* NCCP11945 for New Zealand [14] isolates, and *N. gonorrhoeae* FA1090 *N. gonorrhoeae* reference strain genomes for Saskatchewan [30, 31] and EuroGASP 2013 [18] isolates (see Additional file 1: Table S1). The WHO sequencing read datasets along with reference genomes, Ng plasmid sequences, AMR gene sequences, allelic sequences with their profiles and metadata for NG-MLST and NG-STAR along with locally created databases of Ng plasmid sequences (Additional file 1: Table S2) and MLST allelic sequences are provided in the Gen2Epi VirtualBox image. The NG-MAST alleles datasets are already been provided with the NGMASTER program [13].

#### Pipeline infrastructure

**Fig. 1:**
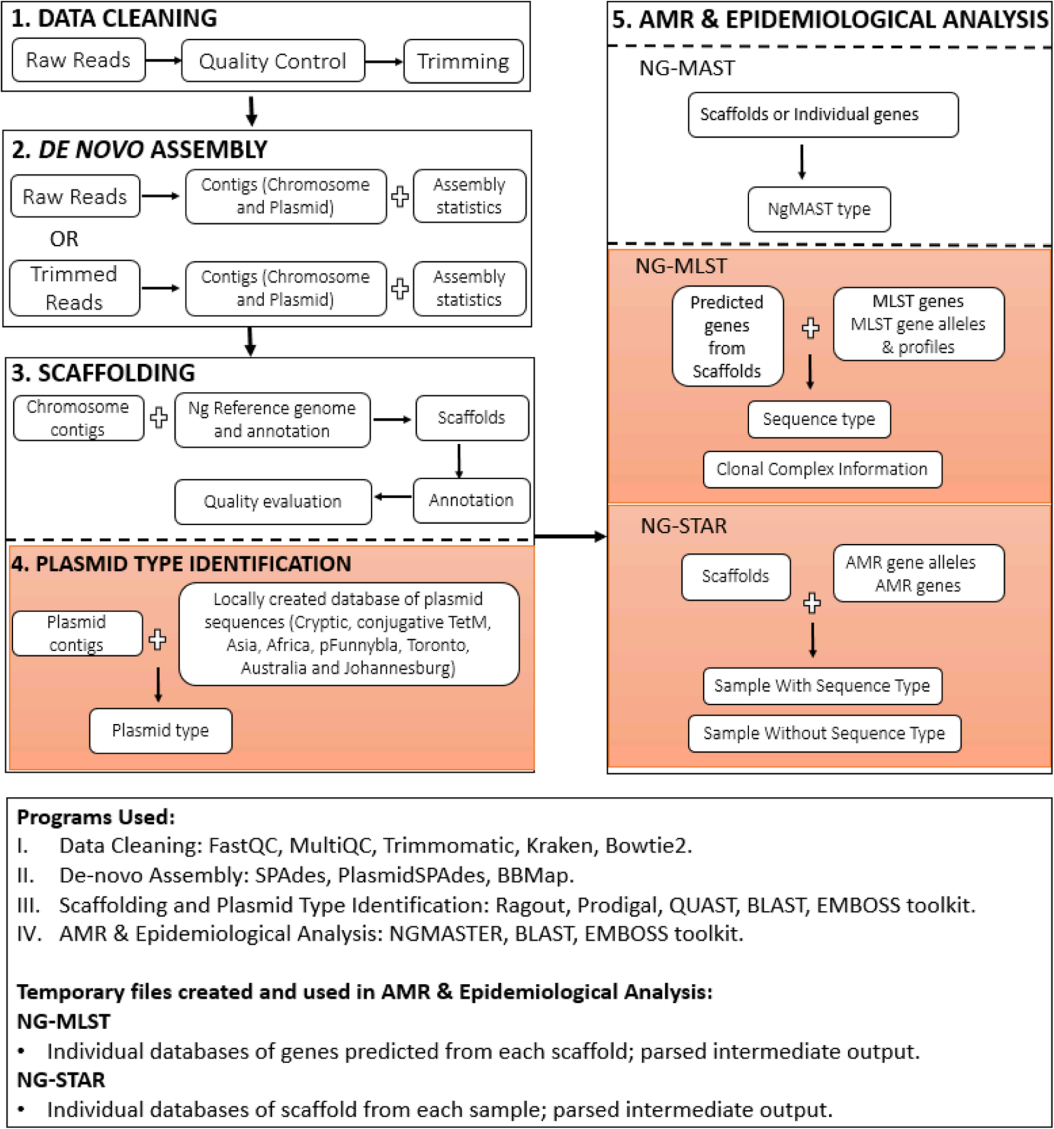
A flowchart outlining the design of the Gen2Epi pipeline with integrated third-party software. Pipeline design sections highlighted in orange color are implemented via custom scripts

Gen2Epi is a Linux-based command-line pipeline providing fully integrated analysis to assemble reads to scaffolds and link the assembled genomes to strain typing (NG-MLST and NG-MAST) and AMR determinant (NG-STAR) information in one place. The pipeline design integrates third-party software (Additional file 1: Table S3) and in-house Perl scripts. All software dependencies are freely available under general public license. Integration of different components is facilitated by using standard input and output formats such as FASTQ, FASTA, plain text, and BLAST report. The architecture of the pipeline has been implemented in five main modules (Fig. 1) consisting of 1) data cleaning; 2) de novo assembly of chromosomes and plasmids; 3) scaffolding, annotation, and quality evaluation; 4) plasmid-type identification (see Additional file 1: Table S2); and 5) molecular epidemiological and AMR determinant prediction. Complete documentation (user manual), along with the pipeline source code can be found at ftp://www.cs.usask.ca/pub/combi or ftp://ftp.cs.usask.ca/pub/combi. Each sequential step of the pipeline are automatic, linked with each other and described below.

#### Step 1: Data Cleaning

Prior to genome assembly, removing adapter sequences and low-quality bases from the raw FASTQ reads is common practice. In Gen2Epi, input raw read datasets in FASTQ format are trimmed to remove low-quality reads (at phred score Q15) using Trimmomatic [34] after an initial quality check using FastQC [35]. Mismatched reads are not currently eliminated. FastQC results for multiple samples (WGS data generated from multiple Ng isolates) are merged into one report using MultiQC [36]. The program does data cleaning automatically in a single step. Furthermore, functions for contamination checks and raw read mapping to the *N. gonorrhoeae* reference genomes are implemented using Kraken [37] and Bowtie2 [38]. The pipeline allows users to customize the combination of these tools applied to clean the raw reads in this step (see Additional file 2: Technical details). For users who do not want to trim the reads, this step can be skipped by directly using the raw dataset in step two for de novo assembly.

#### Step 2: De novo assembly of chromosome and plasmid

In the second stage, the output from step one is used to generate contigs using separate de novo assemblies of chromosomes and plasmids. Raw or trimmed reads are assembled into contigs using SPAdes [18] (“--k 21, 33, 55, 77, 99, 127”, “--pe1–1”, “--pe1–2”, “--careful” and “--cov-cutoff auto”), whereas plasmid reads are assembled into contigs with plasmidSPAdes [39] (SPAdes with parameters “--plasmid”, “--pe1–1”, “--pe1–2”, “--careful” and “--cov-cutoff auto”). In addition, this step also utilizes the “stats.sh” function from BBMap [40] to generate a contig assembly statistics report of the assembled contigs. The resulting contigs can be directly imported into the next step.

#### Step 3: Scaffolding, annotation, and quality evaluation

In this step, assembled contigs (in FASTA format) from step two are joined into full scaffolds by using the reference-based scaffolding approach implemented in Ragout [22]. The resulting scaffolds (FASTA files) are subjected to gene prediction using Prodigal [41]. The quality of the assembled scaffolds is then assessed by comparison with the respective reference genome for potential misassembled and chimeric contigs using QUAST [42]. The completeness of the assembled genomes can also be assessed via optional manual examination of the multiple alignments of scaffolds with the respective *N. gonorrhoeae* reference genome in Mauve [43].

#### Step 4: Plasmid-type Identification

Assembled plasmid contigs generated from step two are aligned against a built-in database of plasmid sequences using NCBI BLASTN [44] to identify the plasmid type. An optional script carries out this step. The built-in database contains the sequences of eight *N. gonorrhoeae* plasmids (including six β-lactamase, conjugative, conjugative tetM, and cryptic plasmids) (see Additional file 1: Table S2).

#### Step 5: Molecular epidemiological analysis and AMR determinant predictions

This step implements the combination of three *N. gonorrhoeae* strain-typing schemes. To achieve this, NG-MAST types are assigned directly from full scaffolds using NGMASTER [13]. In order to characterize strains according to the NG-MLST scheme, a locally created database of annotated genes (created automatically by the script) from each sample is queried using the MLST nucleotide sequences. The resulting MLST genes identified from each scaffold are further used as queries against a built-in database of MLST allelic sequences (from pubMLST on July 4, 2018 [6]) using BLASTN in order to assign allelic types to them. The alignment output is parsed and analyzed, and the sequence type and clonal complex information from a combination of MLST gene alleles from each sample are subsequently retrieved from the MLST profiles.

To perform NG-STAR typing, AMR genes (Additional file 1: Table S2) are extracted by aligning them against a locally created database of individual scaffolds (created automatically by the script) from every sample using BLASTN [44] and functions imported from the EMBOSS toolkit [45]. The respective sequence type (ST) and nomenclature of *N. gonorrhoeae* loci associated with AMR are assigned to each allelic combination by searching against AMR gene profiles and metadata (downloaded from NG-STAR website on July 4, 2018 [10] and provided as part of Gen2Epi).

All three typing schemes (NG-MAST, NG-MLST and NG-STAR), along with alignment and parsing, are carried out using a single custom script. The WHO sequencing read datasets and reference genomes, along with locally created plasmid databases used in step 4, are included within the Gen2Epi distribution.

## Results and discussion

### Gen2Epi assembles short reads into full genomes

The Gen2Epi pipeline successfully assembled the short reads from 1484 *N. gonorrhoeae* WGS samples into full-length genomes. Using the recommended hardware configuration (see Availability and Requirements below), full analysis required approximately 12 h of elapsed time to complete on a laptop with a 3.60 GHz Intel(R) processor. The detailed results of the full genome assembly evaluation in terms of measures such as median misassemblies, alignment length, and different “N” statistics (N50, NA50, NG50, and NGA50 [42]) are shown in Table 1. In contrast to Saskatchewan, New Zealand [14] and EuroGASP 2013 isolates [18], full genomes generated using WHO reference strains had higher NGA50 with no misassemblies, which suggests that, in the case of WHO reference strain isolates [17], the assembled genomes (generated by using trimmed reads) were nearly perfect (Additional file 3: Figure S1). The other striking difference among results generated from the data from these four studies using the Gen2Epi pipeline is that the median genome coverage of full-length scaffolds of WHO isolates [17] was higher (95.95%) than for Saskatchewan [30, 31] (93.88%), New Zealand [14] (92.33%) and EuroGASP 2013 isolates [18] (92.62%). The reason for nearly perfect scaffolds (higher genome coverage) in WHO isolates is the use of existing full reference genomes for each sample.

**Table 1:** Evaluation of the genome assemblies. Measures are median values

	WHO reference strains	Saskatchewan (NML Samples)†	New Zealand	EuroGASP 2013^¥^
# samples	11	27	398	1048
Scaffolds	1	1	1	1
Longest Length	2,167,463	2,210,644	2,212,822	2,212,219
Reference Length	2,172,826	2,232,367	2,232,025	2,153,922
GC (%)	52.64	52.49	52.53	52.52
Misassemblies	0	23	11	11
Unaligned Length	91,387	131,041	153,005	223,989
Genome Fraction (%)	95.95	93.88	92.33	92.62
Duplication ratio	1.01	1.01	1.01	1.01
N’s per 100 kb	4195.81	3873.08	5676.20	5874.67
Indels per 100 kb	1.78	30.72	25.14	30.73
Total aligned length	2,085,686	2,080,404	2,050,100	1,988,450
N50^α^	2,167,463	2,210,644	2,212,822	2,212,219
NA50^α^	2,050,950	225,330	301,699	239,517
NG50^α^	2,167,463	2,210,644	2,212,822	2,212,219
NGA50^α^	2,050,950	223,320	293,413	240,269

**†**In one Saskatchewan sample (32657), a large number of reads were filtered out during the trimming process. This most likely is the reason for the poorer values in this case

^¥^Six samples were excluded from the analysis due to the lack of SRA numbers

^α^N50 is defined as the length of the shortest contig at 50% of the total assembly length. NG50 is similar to N50, except that they are based on genome size rather than assembly size. NA50 and NGA50 are similar to N50 and NG50 except it is based on the alignment of the contigs against a reference genome

To compare the completeness of the genomes generated by Gen2Epi with published results, optimally fully assembled genomes from these previously published studies should be used [14, 17, 18, 30, 31]. However, due to the unavailability of such full genomes (with the exception of a WHO study [17]), we could not make this comparison. In the case of Saskatchewan and New Zealand isolates [30, 31], the assemblies were terminated at the level of contigs. For the EuroGASP 2013 isolates, the authors assembled the reads into multiple scaffolds per genome. The unavailability of these scaffolds as single sequences made it difficult to use them for comparison. However, some comparisons were possible. The “N” statistics values reported for Gen2Epi in the case of the New Zealand samples were higher than previously published values (N50: 40970, NG50: 40198, and NGA50: 35250) [14]. This shows that the use of scaffolding is capable of improving draft genome assemblies generated from short reads. Moreover, the median genome coverage by Gen2Epi in the case of the New Zealand isolates was 92.3%, lower than the previously published result (93.5%) [14]. The probable reason behind this result is that Lee et al. [14] used raw reads to map against the *N. gonorrhoeae* NCCP11945 genome, whereas Gen2Epi aligned scaffolds.

Even though full genomes generated by the proposed pipeline were accurate and overcame the limitation of gene fragmentation over multiple contigs, there were still some irregularities (see Additional file 1: Table S4C where missing AMR genes are represented with “NA”) that can be seen in the results. A characteristic of the pipeline is that it filters out assembled contigs having low-quality bases and contamination during the scaffolding process, occasionally resulting in important AMR genes (such as 23S rRNA) being missing from the molecular marker identification and AMR analysis step. This occurred in the case of the irregularities noted in Table S4C (Additional file 1: Table S4C), supporting the notion that the accuracy and completeness of the final assembled genome is highly dependent upon the quality of the input dataset. Furthermore, we could not compare these irregularities with the previous work as, except for the Saskatchewan isolates [30, 31], none of the published studies [14, 17, 18] used in this analysis performed NG-STAR typing.

Trimming is one of the most effective methods to remove the poor quality bases at the end of the reads from input datasets as error introduced during sequencing cycles by the Illumina sequencing platform often leads to degradation in base quality [46]. The effects of trimming were most apparent in the current analysis when contigs generated from trimmed reads were compared with those generated from raw reads, as in the case of the Saskatchewan and EuroGASP 2013 isolates. A previous study [47] showed that read trimming has effects on genome assembly in terms of average assembly length, largest scaffolds and “N” statistics. We also observed these partial negative effects in one case (sample 32,657 from the Saskatchewan isolates) where a large number of reads were filtered out during the trimming process and consequently very few contigs (# contigs = 3) were assembled. In the case of EuroGASP 2013 isolates, differences were observed in three samples (ERR147119, ERR1560830, and ERR1469562) where STs were different from those previously reported by the authors (see the next section for further explanation).

### Accuracy in automatic assignment of molecular epidemiological markers and AMR determinants

To assess the accuracy of in silico molecular epidemiological markers and AMR determinants generated by the Gen2Epi pipeline, we first analyzed the strain typing results from published studies that have used one or all of the three typing schemes (Table 2). The Saskatchewan study [30, 31] used all three typing schemes, while in the case of New Zealand samples [14], only NG-MAST results were reported. For the original WHO reference strains study, NG-MAST and NG-MLST were performed to characterize them phenotypically [17]. For the EuroGASP 2013 isolates [18], authors performed NG-MAST and NG-MLST typing schemes. Gen2Epi not only generated the same strain type and AMR marker information (with a few minor explainable exceptions – please see next paragraph for details) as reported previously for the 1484 samples but also was able to automatically and simultaneously assign these molecular epidemiological marker results from NG-MLST [5], NG-MAST [11] and NG-STAR [9]. Sample output generated by the Gen2Epi AMR & Molecular Epidemiological Analysis step is shown in Additional file 1: Table S4.

**Table 2:** AMR and molecular epidemiological results

		WHO reference strains	Saskatchewan Isolates^£^	New Zealand Isolates	EuroGASP 2013 isolates^€^
Gen2Epi	NG-MAST	11/11	13/13	57/57	377/377
	NG-MLST	9/9	3/3	NA	102/103

^£^In the case of Saskatchewan isolates, 2 NG-MAST STs and 9 NG-MLST STs were not previously published

^€^The only ST out of 103 NG-MLST sequence types that Gen2Epi could not identify was 1925 because the corresponding sample was not included in the present study

In the case of the Saskatchewan isolates, the NG-STAR results for 12 isolates are 100% identical to the previously published results; information for the remaining 15 isolates is not given in the original publications [30, 31]. For the EuroGASP 2013 isolates (Table 2) [18], using Gen2Epi we were able to assign the isolates to the same 377 NG-MAST sequence types as previously reported [18]. Out of 1048 isolates, 1022 (98%) were exact matches while 26 samples (2%) showed variation. Out of 26 samples, Gen2Epi assigned either an ST number or “new” ST to the 17 isolates that were reported as “multiple” ST in the previous study [18]. Two isolates with a new ST now have a number (one has an ST number [16219] whereas another has a Tbpb allele number [2597]). Gen2Epi was unable to assign STs to the remaining 7 samples (out of 26 isolates) due to missing AMR genes (because of filtering of these genes during the scaffolding process). Moreover, 102 MLST sequence types were identical between the published results of EuroGASP (where a total of 103 MLST STs were reported) and the results produced by Gen2Epi. We observed that three samples (ERR147119, ERR1560830, and ERR1469562) had different STs than previously reported by the authors. To find the reason behind these differences, we reanalyzed these samples directly using raw reads. Consequently, we were able to assign the same ST (assigned in published studies) to sample ERR1469562. However, for samples ERR147119 and ERR1560830, the predicted STs were still different from the published ones. In addition, we observed the presence of “NA” occasionally in the NG-STAR output (Additional file 1: Table S4C) due to the filtering of AMR genes with low-quality bases during the scaffolding process. This finding indicates that the effect of data trimming on genome assemblies should be studied in detail.

#### Gen2Epi pipeline validation

The pipeline was validated by one of the authors (Demczuk) at a separate site (National Microbiology Laboratory, Public Health Agency, Winnipeg) by analyzing  the WHO  *N. gonorrhoeae* reference strains (the WGS isolates are provided in the Gen2Epi VirtualBox image) and obtaining identical results.

### Comparison with existing pipelines and available tools

We compared the Gen2Epi pipeline with existing tools developed using different techniques and exhibiting various features, and which have been widely used by the *N. gonorrhoeae* research community. The detailed comparison is shown in Table 3. Some highlights are as follows.

**Table 3:** Comparison of functions and features of different tools commonly used by the *N. gonorrhoeae* scientific community

	Gen2Epi	Sanger^α^	Nullarbor	Pathogenwatch	pubMLST	NG-MAST	NG-STAR	SRST	NGMASTER	Patric
Data Cleaning	Yes	No	Yes	No	No	No	No	No	No	Yes
Contamination	Yes	No	Yes	No	No	No	No	No	No	Yes
Read Mapping	Yes	No	Yes	No	No	No	No	No	No	No
De novo assembly	Yes	No	Yes	No	No	No	No	No	No	Yes
Scaffolding	Yes	Yes	No	No	No	No	No	No	No	Yes
Plasmid assembly	Yes	No	No	No	No	No	No	No	No	Yes
Plasmid-type identification	Yes	No	No	No	No	No	No	No	No	No
Annotation	Yes	Yes	Yes	No	No	No	Yes	No	No	Yes
MAST	Yes	No	No	Yes	Yes	Yes	No	No	Yes	Yes
MLST	Yes	No	Yes	Yes	Yes	No	No	Yes	No	No
STAR	Yes	No	No	No	Yes	No	Yes	No	No	No
Tet Resistance	Yes	No	Yes	Yes	Yes	No	No	No	No	Yes
Usage	Command-line	Command-line	Command-line	Web-application	Web-application	Web-application	Web-application	Command-line	Command-line	Web-application
Output format	Text, FASTA	FASTA	Tabular, html	Tabular, html	Tabular, html	Tabular, html	Tabular, html	Text	Text	Tabular,html
Language	Perl	Perl	Perl	Unknown	Perl	Unknown	Perl	Python	Python	ECMAScript
Reference		20	29	21	6	12	10	8	13	47

^α^Sanger Institute Assembly Pipeline

Firstly, in terms of functional features, both the Gen2Epi and Nullarbor pipelines can perform data cleaning and contamination checks on the raw reads. For assembly, four tools (Gen2Epi, Nullarbor [29], Patric [48], and Sanger pipeline [20]) can de novo assemble raw reads into contigs. However, only Gen2Epi, Patric, and the Sanger pipeline can perform scaffolding on the assembled contigs. In addition, neither Patric nor the Sanger pipeline contains a read-mapping feature. Five tools (Nullarbor pipeline [29], Pathogenwatch [21], pubMLST [6], Patric [48], and SRST [8]) can identify the NG-MLST strain type from assembled contigs/short-reads/MLST genes. NG-MAST [12], NGMASTER [13], and NG-STAR [10] can extract Multiantigen Sequence Typing (MLST) and AMR determinant information. Therefore, when using the latter existing tools, users have to turn to each alternative tool separately to obtain the strain typing information from their WGS data, but Gen2Epi can directly link all this information in one place.

Secondly, all of these tools fall into two groups based on their user interface. Tools such as Gen2Epi, Sanger pipeline, Nullarbor, SRST, and NGMASTER in the first group have a command-line interface. In the second group, tools such as Patric, pubMLST, NG-MAST, Pathogenwatch, and NG-STAR are web-based applications that are convenient to use but may be frustrating or impractical when users have to deal with a large dataset, especially in cases of manual retrieval of the final outcomes. Taking convenience and flexibility into consideration, Gen2Epi provides complete WGS analysis in five simple steps and provides results in a ready-to-use format.

Thirdly, the input and output formats are quite different among the existing tools and software. For input files, the Nullarbor, Sanger pipeline, and SRST tools typically require a tab-limited text file, genomes in FASTA format, assembly in FASTA format, and reads in a FASTQ format for both forward and reverse orientations. Only FASTQ files are required as an input for Patric. In contrast, all of the three strain typing tools (pubMLST, NG-MAST, and NG-MLST) require AMR genes identified from the assembled contigs in FASTA format. Gen2Epi requires input data in FASTQ (raw or trimmed readsets) and FASTA formats.

Finally, the biggest limitation with both NG-MAST and NG-STAR is that these tools do not use assembled contigs or scaffolds to determine strain typing and AMR determinants. Gen2Epi overcomes this limitation by allowing users to determine strain typing directly from assembled scaffolds and predicted genes. Therefore, Gen2Epi is superior in comparison because it enables users to assemble multiple samples efficiently into scaffolds and assign molecular epidemiological results automatically and simultaneously.

Functions for variant calling, mismatched read elimination, novel AMR determinant prediction, assembly improvement using long reads, and scripts that can take advantage of API (Application Programming Interface) facilities implemented in pubMLST, will be included in the next version. Modules for phylogeny based on core genome analysis [49] will also be implemented in next release as a previous study [50] showed that typing based phylogenetic analysis often leads to a less resolved tree, and that this limitation can be overcome by performing the analysis on a genome-wide scale. In order to make Gen2Epi easy to use for the clinician, a Graphical User Interface (GUI) will be implemented in a future release. It will make use of freely available perl or python libraries so as to be portable and platform independent.

The first two steps (Data Cleaning, De novo assembly of Chromosome and Plasmid, and Plasmid-type Identification) of the current Gen2Epi version are universal for the analysis of other pathogenic bacteria such as *Chlamydia trachomatis*, *N. meningitidis,* and *Mycobacterium tuberculosis*. However, functions for the complete WGS analysis (including novel scaffolding and typing scripts) of other bacteria will be implemented in the future GUI version of Gen2Epi.

## Conclusions

We have developed a novel WGS pipeline named Gen2Epi to assemble Illumina short reads to full genomes and assign strain typing (NG-MAST and NG-MLST) and AMR determinant information (NG-STAR) automatically to the assembled genomes. The accuracy of the pipeline was validated by testing it on 1484 *N. gonorrhoeae* isolates from four previously published studies [14, 17, 18, 30, 31]. Comparison with existing tools indicated that Gen2Epi outperformed in both functional features and handling multiple samples simultaneously. Furthermore, the pipeline works well both with raw reads and trimmed readsets. Gen2Epi is intended to generate strain typing and AMR determinant output results that are easy to handle with less manual effort.

## Availability and requirements

**Project name:** Gen2Epi.

**Project homepage:** Anonymous ftp from URL ftp://www.cs.usask.ca/pub/combi. Any standard web browser can be used to access this site.

**Operating system(s):** Platform Independent.

**Programming language**: Perl.

**Other requirements:** VirtualBox 5.2.14 or higher. The Gen2Epi virtual machine requires a minimum of 29 GB of free hard drive space, 2 GB of RAM, and 2 processors to efficiently run the pipeline on the test dataset included with the software distribution. With this virtual machine configuration on a computer with a 3.6GHz CPU, approximately 12 h of elapsed time are required to complete the full analysis of the test data.

**License:** CC BY-NC 2.0 CA.

## Additional files

Additional file 1: **Table S1.** Datasets used for pipeline testing and evaluation. **Table S2.** Names and accession numbers of input data used by the Gen2Epi pipeline. **Table S3.** List of third-party software incorporated into Gen2Epi. **Table S4.** Sample output generated from Gen2Epi’s AMR & Molecular Epidemiological Analysis step shown in tabular form. (DOCX 25 kb)

Additional file 2: Technical details. (DOCX 20 kb)

Additional file 3: **Figure S1.** Genome alignment of the Gen2Epi-produced WHO G scaffold against the corresponding *Neisseria gonorrhoeae* reference genome using Mauve. The extent of the colored bar indicates the strong similarity between the scaffold and the reference genome. (DOCX 48 kb)

## Abbreviations

AMR: antimicrobial resistance
API: Application Programming Interface
BIGSdb: Bacterial Isolate Genome Sequence Database
ENA: European Nucleotide Archive
MLST: Multilocus Sequence Typing
NCBI: National Center for Biotechnology Information
Ng: *Neisseria gonorrhoeae*
NG-MAST: *N. gonorrhoeae* Multiantigen Sequence Typing
NG-STAR: *Neisseria gonorrhoeae* Sequence Typing for Antimicrobial Resistance
SRST: Short Read Sequence Typing
ST: Sequence Type
STI: sexually transmitted infection
WGS: whole genome sequencing
WHO: World Health Organization
