# Supplementary material for: Gen2Epi: an automated whole-genome sequencing pipeline for linking full genomes to antimicrobial susceptibility and molecular epidemiological data in Neisseria gonorrhoeae
Source: BMC Genomics. 2019 Mar 4;20:165. doi: 10.1186/s12864-019-5542-3 (PMC6398234; doi:10.1186/s12864-019-5542-3)
Supplement: Supplementary file 1 — Table S1. Datasets used for pipeline testing and evaluation. Table S2. Names and accession numbers of input data used by the Gen2Epi pipeline. Table S3. List of third-party software incorporated into Gen2Epi. Table S4. Sample output generated from Gen2Epi’s AMR & Molecular Epidemiological Analysis step shown in tabular form. (DOCX 25 kb) [file 12864_2019_5542_MOESM1_ESM.docx]

**Table S1: Datasets used for pipeline testing and evaluation**

| **Study** | **Sequencing platform** | **# Samples** | **Reference genome used** | **Ref** |
| --- | --- | --- | --- | --- |
| WHO Reference Strains | Illumina HiSeq | 11 | F (LT591897), G (LT591898), K (LT591908), L (LT591901), M (LT591904), N (LT591910), O (LT592146), P (LT592157), X (LT592155), Y (LT592161), Z (LT592153).  ENA BioProject accession no: PRJEB14020 | 17 |
| Saskatchewan isolates^†^ | Illumina MiSeq | 27 | FA1090 (NC_002946) | 30-31 |
| New Zealand | Illumina NextSeq | 398 | NCCP11945 (NC_011035)  ENA BioProject accession no: PRJNA394216 | 14 |
| EuroGASP Collection | Illumina HiSeq | 1048 | NCBI SRA accession no: ERR1680294^£^ | 18 |
| **Total** | | **1484** | | |

^†^The raw read datasets (“Raw_Data.tar.gz”) for Saskatchewan isolates are available at <ftp://www.cs.usask.ca/pub/combi>

^£^ In order to compare the results generated by Gen2Epi and those published by Harris et al. [18], accession numbers of samples (1054 WGS read datasets downloaded from NCBI [33]) were matched with the EuroGASP collection (1048 genomes) available at Pathogenwatch (<https://pathogen.watch/>). Six samples (ECDC_RB13001545, ECDC_RB13001559, ECDC_RB13001584, ECDC_RB13001585, ECDC_RB13000575, and ECDC_RB13000574) were excluded from the analyses due to the absence of SRA accession numbers.

**Table S2: Names and accession numbers of input data used by the Gen2Epi pipeline**

|  | **NCBI accession numbers** |
| --- | --- |
| Plasmids | Cryptic = NC_001377.1, Conjugative = CP020416.2, Conjugative tetM = NC_014105.1, Asia-type^1^ = NC_002098.1, Africa-type^1^ = MH140435, pFunnybla = MH140434, Toronto-type^2^ = NC_010881.1, Australia-type^3^ = NC_025191.1, and Johannesburg-type^4^ = NC_019211.1 |
| NG-MLST genes | *abcZ* = AJ242840, *adk* = L36471.1, *aroE* = U82842.1, *fumC* = AY522859.1, *gdh* = FMTD01000035.1^†^ (complement [14282..15727]), *pdhC* = FMTC01000001.1^†^ (complement [44907..47570]), and *pgm* = U02489.1 |
| NG-STAR genes | *porB* = Z69259.1, *penA* = M32091.1, *mtrR* = KT954119.1, *ponA* = AB727718.1, *gryA* = U08817.1, *parC* = U08907.1, and *23S* = X67293.1 |

^†^Due to the availability of only the partial CDS in the case of *gdh* and *pdhC*, the respective full nucleotide sequences were extracted from whole genome assemblies.

**References:**

1. Pagotto F, Aman AT, Ng LK, Yeung KH, Brett M, and Dillon JA. Sequence Analysis of the Family of Penicillinase-Producing Plasmids of *Neisseria gonorrhoeae*. Plasmid 2000; **43**: 24–34.
2. R Scharbaai‐Vázqueza, T Candelas, Torres‐Bauzá LJ. Mobilization of the Gonococcal 5.2 kb Beta‐Lactamase Plasmid pSJ5.2 into *Escherichia coli* by Cointegration with Several Gram-Conjugative Plasmids. Plasmid 2007; **57**: 156–64.
3. Trembizki E, Buckley C, Lawrence A, Lahra M, Whiley D, and GRAND Study Investigation. Characterization of a Novel *Neisseria gonorrhoeae* Penicillinase-Producing Plasmid Isolated in Australia in 2012. Antimicrob Agents Chemother 2014; **58**: 4984–5.
4. Muller EE, Fayemiwo SA, Lewis DA. Characterization of a Novel β-Lactamase-Producing Plasmid in *Neisseria gonorrhoeae*: Sequence Analysis and Molecular Typing of Host Gonococci. J Antimicrob Chemother 2011; **66**: 1514–7.

**Table S3: List of third-party software incorporated into Gen2Epi.**

| **Program Name** | **Version** | **Source Website** | **Ref** |
| --- | --- | --- | --- |
| FastQC | 0.11.7 | <https://www.bioinformatics.babraham.ac.uk/projects/fastqc/fastqc_v0.11.7.zip> | 35 |
| MultiQC | 1.5 | <https://github.com/ewels/MultiQC> | 36 |
| Trimmomatic | 0.33.0 | <https://github.com/timflutre/trimmomatic> | 34 |
| Kraken | 1.1 | <http://ccb.jhu.edu/software/kraken/> | 37 |
| Bowtie2 | 2.3.4.1 | <https://sourceforge.net/projects/bowtie-bio/files/bowtie2/2.3.4.1> | 38 |
| SPAdes | 3.12.0 | <http://cab.spbu.ru/files/release3.12.0/SPAdes-3.12.0-Linux.tar.gz> | 19 |
| BBMap | 38.08 | <https://sourceforge.net/projects/bbmap/> | 40 |
| Ragout | 2.0 | <https://github.com/fenderglass/Ragout> | 22 |
| Prodigal | 2.6.3 | <https://github.com/hyattpd/Prodigal> | 41 |
| QUAST | 5.0.0 | <https://sourceforge.net/projects/quast/> | 42 |
| BLAST | 2.7.1+ | <https://ftp.ncbi.nlm.nih.gov/blast/executables/blast+/2.7.1/> | 44 |
| EMBOSS | 6.5.7 | <ftp://emboss.open-bio.org/pub/EMBOSS/old/6.5.0/> | 45 |
| NGMASTER | 0.5.1 | <https://github.com/MDU-PHL/ngmaster> | 13 |

**Table S4: Sample output generated from Gen2Epi’s AMR & Molecular Epidemiological Analysis step shown in tabular form**

1. **NG-MAST**

|  | **POR** | **TBPB** | **NG-MAST ST** |
| --- | --- | --- | --- |
| WHO-F | 2002 | 165 | 3303 |
| WHO-G | 90 | 18 | 621 |
| WHO-K | 917 | 10 | 1424 |
| WHO-L | 915 | 21 | 1422 |
| WHO-M | 2000 | 4 | 3304 |
| WHO-N | 90 | 122 | 556 |
| WHO-O | 91 | 32 | 495 |
| WHO-P | 2001 | 29 | 3305 |
| WHO-X | 2594 | 10 | 4220 |
| WHO-Y | 908 | 110 | 1407 |
| WHO-Z | 1059 | 10 | 4015 |

1. **NG-MLST**

|  | **abcZ** | **adk** | **aroE** | **fumC** | **gdh** | **pdhC** | **pgm** | **NG-MLST ST** |
| --- | --- | --- | --- | --- | --- | --- | --- | --- |
| WHO-F | 200 | 39 | 67 | 157 | 148 | 153 | 65 | 10934 |
| WHO-G | 126 | 39 | 67 | 157 | 148 | 153 | 65 | 1903 |
| WHO-K | 59 | 39 | 67 | 78 | 148 | 153 | 65 | 7363 |
| WHO-L | 126 | 39 | 67 | 78 | 149 | 153 | 65 | 1590 |
| WHO-M | 109 | 39 | 67 | 111 | 148 | 153 | 133 | 7367 |
| WHO-N | 59 | 39 | 67 | 111 | 148 | 153 | 65 | 1583 |
| WHO-O | 109 | 39 | 170 | 158 | 148 | 153 | 65 | 1902 |
| WHO-P | 126 | 39 | 67 | 111 | 149 | 153 | 133 | 8127 |
| WHO-X | 59 | 39 | 67 | 78 | 148 | 153 | 65 | 7363 |
| WHO-Y | 109 | 39 | 170 | 111 | 148 | 153 | 65 | 1901 |
| WHO-Z | 59 | 39 | 67 | 78 | 148 | 153 | 65 | 7363 |

1. **NG-STAR**

|  | **penA** | **penA**  **Markers** | **mtrR** | **mtrR**  **Markers** | **porB** | **porB**  **Markers** | **ponA** | **ponA**  **Markers** | **gyrA** | **gyrA**  **Markers** | **parC** | **parC**  **Markers** | **23S** | **23S**  **Markers** |
| --- | --- | --- | --- | --- | --- | --- | --- | --- | --- | --- | --- | --- | --- | --- |
| WHO-F | 15.001 | Type XV Non-Mosaic | 14 |  | 14 | porB1a | 0 | Wild Type | 0 | Wild Type | 7 | Wild Type | 0 | Wild Type |
| WHO-G | 2.001 | Type II Non-Mosaic | 1 | -35A Del | 13 | porB1a | 1 | L421P | 5 | S91F | 1 | Wild Type | 0 | Wild Type |
| WHO-K | 10.001 | Type X Mosaic | 19 | -35A Del, G45D | 8 | G120K, A121D | 1 | L421P | 2 | S91F, D95N | 5 | S87R, S88P | NA^†^ | NA^†^ |
| WHO-L | 7.001 | Type VII Non-Mosaic | 25 | G45D | 8 | G120K, A121D | 1 | L421P | NA^†^ | NA^†^ | 11 | D86N, S88P | 0 | Wild Type |
| WHO-M | 2.001 | Type II Non-Mosaic | 19 | -35A Del, G45D | 8 | G120K, A121D | 1 | L421P | 1 | S91F, D95G | NA^†^ | NA^†^ | 0 | Wild Type |
| WHO-N | 2.001 | Type II Non-Mosaic | 26 | A39T | 13 | porB1a | 1 | L421P | 1 | S91F, D95G | 12 | S87I | 0 | Wild Type |
| WHO-O | 12.001 | Type XII Non-Mosaic | 1 | -35A Del | 8 | G120K, A121D | 1 | L421P | 0 | Wild Type | 7 | Wild Type | 0 | Wild Type |
| WHO-P | 2.001 | Type II Non-Mosaic | 32 |  | 2 | A121D | 0 | Wild Type | 0 | Wild Type | 0 | Wild Type | 0 | Wild Type |
| WHO-X | 37.001 | Type 37 Mosaic | 1 | -35A Del | 8 | G120K, A121D | 1 | L421P | 2 | S91F, D95N | 5 | S87R, S88P | 0 | Wild Type |
| WHO-Y | 42.001 | Type 42 Mosaic | 1 | -35A Del | 11 | G120K, A121N | 1 | L421P | 1 | S91F, D95G | 3 | S87R | NA^†^ | NA^†^ |
| WHO-Z | 64.001 | Type 64 Mosaic | 51 | Wild Type | 8 | G120K, A121D | 1 | L421P | 2 | S91F, D95N | 5 | S87R, S88P | 0 | Wild Type |

^†^ NA is arbitrarily assigned to the samples where Gen2Epi was unable to find the AMR genes
