# Supplementary material for: Gen2Epi: an automated whole-genome sequencing pipeline for linking full genomes to antimicrobial susceptibility and molecular epidemiological data in Neisseria gonorrhoeae
Source: BMC Genomics. 2019 Mar 4;20:165. doi: 10.1186/s12864-019-5542-3 (PMC6398234; doi:10.1186/s12864-019-5542-3)
Supplement: Supplementary file 2 — Technical details. (DOCX 20 kb) [file 12864_2019_5542_MOESM2_ESM.docx]

**Additional file 2: Technical details**

**Step 1: Data Cleaning**

Input raw read datasets in FASTQ format were trimmed in Gen2Epi to remove low-quality reads using the following command:

*“perl WGS_SIBP_P1.pl <Input> <path-to-fastq-files> trimming <leading length> <trailing length> <sliding window> <minimum length>”*

**Command line arguments:**

WGS_SIBP_P1.pl = name of the script that does the data cleaning.

<Input> =A tab-limited input file giving full sample names and the associated paired-end read files in three columns. An example line in the file might be
“WHO-F WHO-F_S2_L001_R1_001.fastq.gz WHO-F_S2_L001_R1_001.fastq.gz”.
Replace <Input> with the actual filename.

<path-to-fastq-files> = Path of the folder/directory that has all the raw reads as FASTQ files. Replace <path-to-fastq-files> with the actual path.

trimming = Keyword used to direct the program to trim the raw read sets.
<leading length> = Maximum number of bases to remove from the start of a read while base quality is below a threshold.

<trailing length> = Maximum number of bases to remove from the end of a read while base quality is below a threshold.

<sliding window> = Number of bases to average across to calculate average quality and the average quality required, separated by a colon (‘:’)
<minimum length> = A threshold used for filtering reads. All reads must be at least this value in length, otherwise they are removed.

**An example run:** *“perl WGS_SIBP_P1.pl Input WHO_Data trimming 3 3 4:15 30”*

**Quality threshold:**

A phred score of 15 was used during the evaluation of Gen2Epi. In our analysis, we observed that trimming at Q15 greatly reduced the number of reads in one out of 1484 samples (Please see “Gen2Epi assembles short reads into full genomes” under “Results and Discussion” in the main manuscript for further explanation). Our experience is that trimming at phred score 15 is generally applicable to most WGS samples and recommend it as an initial threshold value. We also recommend that users look at the read binning results to make sure that the input reads are free from contamination (reads from other organisms).

**Gen2Epi Usage:**

Gen2Epi is capable of performing a complete WGS analysis starting from data cleaning to AMR identification in five simple steps (see “Pipeline Infrastructure” in the main manuscript). For step1 and 2, users can provide input data in either FASTA or FASTQ format. Furthermore, users can access the pipeline at any stage. For instance, it is possible to use Gen2Epi starting at stage 3 (scaffolding, annotation, and quality evaluation) if users have the assembled contigs in FASTA format. However, in order to use the stage 5, users have to provide scaffolds or predicted nucleotide genes generated from stage 3 in FASTA format.
