# Supplementary material for: Gen2Epi: an automated whole-genome sequencing pipeline for linking full genomes to antimicrobial susceptibility and molecular epidemiological data in Neisseria gonorrhoeae
Source: BMC Genomics. 2019 Mar 4;20:165. doi: 10.1186/s12864-019-5542-3 (PMC6398234; doi:10.1186/s12864-019-5542-3)

**Figure S1**: Genome alignment of the Gen2Epi-produced WHO G scaffold against the corresponding *Neisseria gonorrhoeae* reference genome using Mauve. The extent of the colored bar indicates the strong similarity between the scaffold and the reference genome.


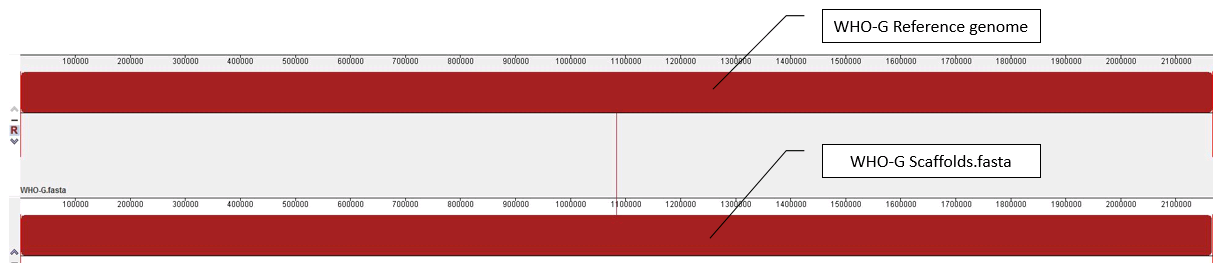

Supplement: Supplementary file 3 — Figure S1. Genome alignment of the Gen2Epi-produced WHO G scaffold against the corresponding Neisseria gonorrhoeae reference genome using Mauve. The extent of the colored bar indicates the strong similarity between the scaffold and the reference genome. (DOCX 48 kb) [file 12864_2019_5542_MOESM3_ESM.docx]
